# Synthesis and characterization of novel iminobenzoates with terminal pyrazine moieties

**DOI:** 10.1186/s13065-018-0396-3

**Published:** 2018-03-08

**Authors:** Mushtaq Ahmad, Zahida Perveen, Adailton J. Bortoluzzi, Shahid Hameed, Muhammad R. Shah, Muhammad Tariq, Ghias ud Din, Muhammad T. Jan, Muhammad Siddique, Muhammad Anwar

**Affiliations:** 1 Medicinal Botanic Centre PCSIR Labs Complex, University Road, Peshawar, 25120 Pakistan; 2 Institute of Chemical Sciences, University of Peshawar, Peshawar, 25120 Pakistan; 3 Departmento de Química, Universidade Federal de Santa Catarina, Florianópolis, SC 88040-900 Brazil; 4 Deparment of Chemistry, Quaid-I-Azam University, Islamabad, 45320 Pakistan; 5 HEJ Research Institute of Chemistry University of Karachi, Karachi, 75270 Pakistan; 6 Department of Chemistry, Shaheed Benazir University, Sheringal, Upper Dir, 18050 KPK Pakistan; 7 Department of Chemistry, Islamia College University, Peshawar, 25120 Pakistan; 8 Present Address: Fujian Institute of Research on the Structure of Matter, Chinese Academy of Sciences, 155 Yangqiao Road West, Fuzhou, 350002 China

**Keywords:** Pyrazine, Pyrazine-2-carbohydrazide, 5-Methylpyrazine-2-carbohydrazide, Triethyl amine, Iminobenzoates, X-ray crystallography

## Abstract

Apart from its numerous biological activities like antidiabetic, anti-inflammatory, antimicrobial, pyrazine moiety plays an important role in luminescent materials. Its role in luminescent materials is due to its highly electron deficient nature specially when it is in the centre along the mainstay of extended π-conjugated systems. Similarly, new liquid crystalline compounds are being made constantly where the central benzoaromatic moiety is being replaced with the heterocycles including pyrazine due to their more variable nature. Pyrazine derivatives can also be used in supramolecular assemblies due to their efficient hydrogen bonding, protonation and complexation properties. Keeping in view the enormous applications of pyrazine derivatives we planned to synthesize new extended iminobenzoates with pyrazine moieties at the terminal positions. The planned iminobenzoates with terminal pyrazine moieties were prepared following standard procedures. The pyrazine-2-carbohydrazide (**1**) and 5-methylpyrazine-2-carbohydrazide (**2**) were prepared by refluxing their methyl esters with hydrazine hydrate in methanol. The esters (**3a**–**3f**) were synthesized by reacting 4-hydroxybenzaldehyde with differently substituted acid halides in tetrahydrofuran in the presence of triethyl amine. The target compounds that is, iminobenzoates with the pyrazine moieties at terminal positions (**4a**–**4l**), were obtained in good to excellent yields by the reaction of the hydrazides with the esters at reflux. The synthesized compounds were fully characterized using different spectroanalytical techniques including FT-IR, NMR, Mass, elemental analysis and single crystal X-ray diffraction analysis. The paper describes the synthesis of novel iminobenzoates following easy methods while utilizing commercially available starting materials. The synthesized iminobenzoates may possibly be converted to compounds with luminescent and liquid crystalline properties after making suitable changes to the pyrazine moieties. Properly substituted pyrazines on both sides, capable of further suitable extensions, may result in compounds with such properties. 
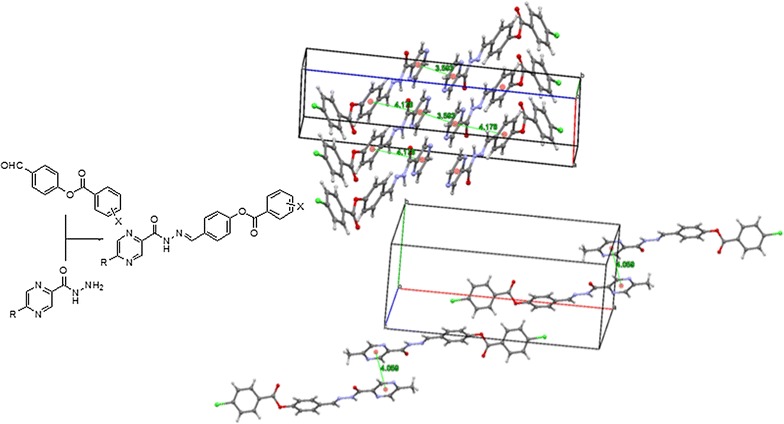

## Introduction

Pyrazine belongs to the six membered heterocyclic diazines with two nitrogen in the same ring at 1, 4 positions, the other members being the pyridazine and pyrimidine with the two nitrogens at 1, 2 and 1, 3 positions respectively [1–4]. Another pyrazine containing heterocycle is the quinoxaline or benzopyrazine. Both pyrazine and quinoxaline derivatives are quite important due to their crucial roles in natural and synthetic compounds [5–10].

Apart from their other bioactivities like antidiabetic [11], anti-inflammatory [12], antimicrobial [13] and diuretic [14], pyrazine derivatives, like pyrazinamide, have a vital role in controlling tuberculosis [15]—a life threatening disease.

Due to their enormous use in a variety fields of day-to-day life, chemistry and medicine, luminescent materials are becoming more and more important continuously. As a result, the importance of the synthesis of the extended π-conjugated systems is increasing day by day as these materials impart immensely useful properties to the potentially used electrooptical (EO) and non-linear optical (NLO) materials in optical technologies. Due to its highly π-electron deficient nature, pyrazine can be used as electron-withdrawing part in push–pull system-the system consisting of two parts-an electron withdrawing and an electron donating part both interlinked via another π-conjugated moiety. This push–pull property of extended π-conjugated systems makes the organic materials as luminescent materials, semiconductors and also makes their use in optical data processing technologies. Pyrazines, which are extremely π-deficient aromatic heterocycles, after playing its part in the push–pull system as dipolar moiety result in intramolecular charge transfer (ICT). This highly important intramolecular charge transfer (ICT) property along the mainstay of the molecule can result in the luminescence properties of the molecule. Due to their efficient hydrogen bonding, protonation and complexation, pyrazine derivatives can be used in supramolecular assemblies forming sensors as well.

Liquid crystal displays are well known alternatives to the cathode ray tube in the market today. Though they will still be the main dominating technology, in at least near future, yet continuous efforts in terms of productive research are to be made to enhance and extend their applications further in a number of demanding displays. For this reason, new liquid crystals with diverse structural features need to be synthesized so as to cope with the challenging market demands for liquid crystal displays. Being more variable than benzoaromatics, heterocyclic compounds are thought to result in a range of useful liquid crystalline compounds after replacing the central benzoaromatic moiety with these heterocycles. Liquid crystals with pyrazine moieties have already been reported [16–19]. Studies on a number of pyrazine containing compounds have revealed that compounds having 2,5-disustituted pyrazines are liquid crystals while those with 1,5-disubstituted pyrazines are not [20].

In continuation to our interests in the synthesis of pyrazine compounds [21–24] and the enormous applications of pyrazine derivatives in a number of fields prompted us to synthesize new extended iminobenzoates with pyrazine moieties at the terminal positions. The study may provide a base to other researchers in the field to expand these studies in different directions for practical use in a variety of fields.

Of the two general methods for the preparation of extended π-conjugated pyrazine systems [25–29] we selected the one involving the derivatization of the easily available starting materials that is, differently substituted pyrazine moieties.

## Materials and methods

### General

Reagents and solvents used were of analytical grade. Melting points were determined via Bock-monoscop-M melting point apparatus. IR spectra were recorded on IRPrestige-21 spectrophotometer (Shimadzu) in the range of 4000–400 cm^−1.^ The NMR spectra were either measured on Avance 300 MHz NMR Spectrometer (Bruker) or Avance 400 MHz NMR Spectrometer (Bruker) in deuterated solvents. Chemical shift values are being reported in ppm (parts per million). Designations to the aromatic protons in the intermediate compounds (**3a**–**3f**) were made as; protons *ortho* to the aldehydic group were designated as 1, 1′ while *meta* as 2,2′; protons from the other aromatic ring (from acid halide) were designated in a clockwise manner throughout with the letters 3, 4, 5 and 6. These designations were maintained in the final products (**4a**–**4l**) as well for easy understanding. Mass spectra were recorded using JEOL JMS 600-H machine. For elemental analysis, Vario EL III CHNS-O Elemental Analyzer was used. X-ray diffraction analyses were carried out with a Bruker APEX II DUO diffractometer using graphite-monochromated MoKα radiation (0.71073 Å) from a sealed tube operating at 50 kV and 30 mA. Temperature of the sample was set at 200 (± 2) K with an Oxford Instruments Cryojet system 700 series. Images were recorded by phi and omega scans using APEX2 software [30]. All collected data were corrected for Lorentz, polarization effects and for absorption. The structures were solved by direct methods and refined applying the full-matrix least-squares on F^2^ method using SHELXS and SHELXL2014 [31] software, respectively. ORTEP plots were drawn with the program PLATON [32]. All non-hydrogen atoms were refined with anisotropic displacement parameters. Hydrogen atoms were placed at their idealized positions with distances of 0.95 Å for C–H_Ar_. The U_iso_ values for the hydrogen atoms were fixed at 1.2 times the U_eq_ of the carrier atom (C). Hydrogen atoms of the N–H groups were found from Fourier difference map and treated with riding model. Full crystallographic tables (including structure factors) for compounds **4d** and **4j** have been deposited with the Cambridge Crystallographic Data Centre as supplementary publication numbers CCDC 1579201–1579202. These data can be obtained free of charge from the Cambridge Crystallographic Data Centre via http://www.ccdc.cam.ac.uk/data_request/cif.

### Synthesis of pyrazine-2-carbohydrazides (**1** and **2**)

Pyrazine-2-carbohydrazide (**1**) and 5-methylpyrazine-2-carbohydrazide (**2**) were prepared following the literature known procedure [24].

### General procedure for the synthesis of esters (**3a**–**3f**)

Aldehyde (8.0 mmol) was dissolved in tetrahydrofuran (40 mL) and triethyl amine (24.0 mmol) was added to it. The mixture was stirred for 15 min and then kept in an ice bath. Acid halide (8.0 mmol) dissolved in tetrahydrofuran (40 mL) was added dropwise to the reaction mixture. Reaction was stirred for 2 h and then filtered. The filtrate was concentrated and the residue was recrystallized from chloroform in petroleum ether.

#### 4-Formylphenyl 2-fluorobenzoate (**3a**)

Colour: off-white solid; yield: 0.76 g, 3.1 mmol, 39%; R_f_: 0.45 (40% acetone in *n*-hexane); mp 145–146 °C; IR ($\bar{\nu }$, cm^−1^): 1745, 1699, 1253, 1207; ^1^H NMR (400 MHz, CDCl_3_): δ 7.19–7.24 (1H, m, H-5), 7.27–7.30 (1H, m, H-3), 7.41–7.43 (2H, m, H-2,2′), 7.60–7.63 (1H, m, H-4), 7.95–7.97 (2H, m, H-1,1′), 8.08–8.11 (1H, m, H-6), 10.01 (1H, s, C*H*O).

#### 4-Formylphenyl 2-chlorobenzoate (**3b**)

Colour: off-white solid; yield: 1.67 g, 6.4 mmol, 80%; R_f_: 0.45 (40% acetone in *n*-hexane); mp 92–93 °C; IR ($\bar{\nu }$, cm^−1^): 1739, 1695, 1253, 744; ^1^H NMR (300 MHz, CDCl_3_): δ 7.41–7.49 (3H, m, H-3,4,5), 7.55–7.57 (2H, m, H-2,2′), 7.98–8.03 (2H, m, H-1,1′), 8.07–8.10 (1H, m, H-6), 10.05 (1H, s, C*H*O).

#### 4-Formylphenyl 3-chlorobenzoate (**3c**)

Colour: off-white solid; yield: 1.0 g, 3.8 mmol, 48%; R_f_: 0.45 (40% acetone in *n*-hexane); mp 97–99 °C; IR ($\bar{\nu }$, cm^−1^): 1728, 1699, 1253, 732; ^1^H NMR (300 MHz, CDCl_3_): δ 7.40 (2H, d, *J* = 8.4 Hz, H-2,2′), 7.47–7.49 (1H, m, H-5), 7.61–7.64 (1H, m, H-4), 7.97 (2H, d, *J* = 8.4 Hz, H-1,1′), 8.06–8.09 (1H, m, H-6), 8.17 (1H, s, H-3), 10.02 (1H, s, C*H*O).

#### 4-Formylphenyl 4-chlorobenzoate (**3d**)

Colour: white crystals; yield: 1.57 g, 6.0 mmol, 75%; R_f_: 0.45 (40% acetone in *n*-hexane); mp 116–118 °C; IR ($\bar{\nu }$, cm^−1^): 1728, 1683, 1261, 746; ^1^H NMR (300 MHz, CDCl_3_): δ 7.43 (2H, d, *J* = 8.7 Hz, H-4,5), 7.53 (2H, d, *J* = 8.7 Hz, H-3,6), 8.00 (2H, d, *J* = 8.4 Hz, H-2,2′), 8.16 (2H, d, *J* = 8.4 Hz, H-1,1′), 10.05 (1H, s, C*H*O).

#### 4-Formylphenyl 3-bromobenzoate (**3e**)

Colour: off-white solid; yield: 1.15 g, 3.8 mmol, 47%; R_f_: 0.45 (40% acetone in *n*-hexane); mp 98–100 °C; IR ($\bar{\nu }$, cm^−1^): 1728, 1697, 1253, 513; ^1^H NMR (400 MHz, CDCl_3_): δ 7.40 (2H, d, *J* = 8.4 Hz, H-2,2′), 7.41–7.42 (1H, m, H-5), 7.77–7.79 (1H, m, H-4), 7.97 (2H, d, *J* = 8.4 Hz, H-1,1′), 8.11–8.13 (1H, m, H-6), 8.33 (1H, s, H-3), 10.02 (1H, s, C*H*O).

#### 4-Formylphenyl 4-bromobenzoate (**3f**)

Colour: off-white solid; yield: 1.76 g, 5.8 mmol, 72%; R_f_: 0.45 (40% acetone in *n*-hexane); mp 172–174 °C; IR ($\bar{\nu }$, cm^−1^) 1741, 1699, 1265, 520; ^1^H NMR (400 MHz, CDCl_3_): δ 7.39 (2H, d, *J* = 8.4 Hz, H-2,2′), 7.66 (2H, d, *J* = 8.4 Hz, H-4,5), 7.96 (2H, d, *J* = 8.4 Hz, H-3,6), 8.05 (2H, d, *J* = 8.4 Hz, H-1,1′), 10.01 (1H, s, C*H*O).

### General procedure for the synthesis of iminobenzoates (**4a**–**4l**)

The hydrazide (3.00 mmol) was dissolved in methanol (50 mL) and added dropwise to a methanolic (50 mL) solution of the ester (3.00 mmol). Reaction mixture was refluxed for 5 h. The solid formed was filtered, washed with cold methanol, dried over anhydrous CaCl_2_ under vacuum and recrystallized from chloroform in *n*-hexane.

#### 4-[(E)-(Pyrazine-2-carboylimino)methyl]phenyl 2-fluorobenzoate (**4a**)

Colour: white shiny crystals; yield: 0.6 g, 1.6 mmol, 56%; R_f_: 0.3 (40% acetone in *n*-hexane); mp 281–290 °C; IR ($\bar{\nu }$, cm^−1^): 3300, 1728, 1674, 1600, 1290, 1228, 1018; ^1^H NMR (300 MHz, DMSO): δ 7.41–7.48 (4H, m, H-1,1′,2,2′), 7.76–7.87 (3H, m, H-3,4,5), 8.09–8.15 (1H, m, H-6), 8.68 (1H, s, *H*C=N), 8.80 (1H, d, *J* = 2.4 Hz, H-5 pyrazine), 8.93 (1H, d, *J* = 2.4 Hz, H-6 pyrazine), 9.28 (1H, s, H-3 pyrazine), 12.36 (1H, s, CON*H*); MS (EI, *m*/*z*): 364 [M^+^], 243, 123, 109, 81, 61.

#### 4-[(E)-(Pyrazine-2-carboylimino)methyl]phenyl 2-chlorobenzoate (**4b**)

Colour: white shiny flakes; yield: 0.9 g, 2.4 mmol, 80%; R_f_: 0.82 (50% acetone in *n*-hexane); mp 262–265 °C; IR ($\bar{\nu }$, cm^−1^): 3288, 1743, 1674, 1560, 1244, 1199, 1020, 750; ^1^H NMR (400 MHz, DMSO): δ 7.44 (2H, d, *J* = 8.4 Hz, H-2,2′), 7.54–7.58 (1H, m, H-3), 7.84–7.86 (2H, m, H-4,5), 7.85 (2H, d, *J* = 8.4 Hz, H-1,1′), 8.10–8.12 (1H, m, H-6), 8.69 (1H, s, *H*C=N), 8.80 (1H, d, *J* = 2.4 Hz, H-5 pyrazine), 8.93 (1H, d, *J* = 2.4 Hz, H-6 pyrazine), 9.27 (1H, s, H-3 pyrazine), 12.33 (1H, s, CON*H*); MS (EI, *m*/*z*): 380 [M^+^], 139, 123, 111, 75, 52.

#### 4-[(E)-(Pyrazine-2-carboylimino)methyl]phenyl 3-chlorobenzoate (**4c**)

Colour: Lemon green powder; yield: 1.0 g, 2.6 mmol, 89%; R_f_: 0.41 (40% acetone in *n*-hexane); mp 265–272 °C; IR ($\bar{\nu }$, cm^−1^): 3302, 1728, 1678, 1610, 1261, 1020, 736; ^1^H NMR (400 MHz, DMSO): δ 7.44 (2H, d, *J* = 8.4 Hz, H-2,2′), 7.64–7.68 (1H, m, H-3), 7.83–7.85 (3H, m, H-4,5,6), 8.10 (2H, d, *J* = 8.4 Hz, H-1,1′), 8.69 (1H, s, *H*C=N), 8.80 (1H, d, *J* = 2.4 Hz, H-5 pyrazine), 8.93 (1H, d, *J* = 2.4 Hz, H-6 pyrazine), 9.27 (1H, s, H-3 pyrazine), 12.32 (1H, s, CON*H*); MS (EI, *m*/*z*): 380 [M^+^], 139, 123, 111, 80, 52.

#### 4-[(E)-(Pyrazine-2-carboylimino)methyl]phenyl 4-chlorobenzoate (**4d**)

Colour: white shiny crystals; yield: 0.95 g, 2.5 mmol, 84%; R_f_: 0.4 (40% acetone in *n*-hexane); mp 287–295 °C; IR ($\bar{\nu }$, cm^−1^): 3292, 1732, 1674, 1591, 1253, 1197, 1012, 738; ^1^H NMR (400 MHz, DMSO): δ 7.42 (2H, d, *J* = 8.4 Hz, H-2,2′), 7.70 (2H, d, *J* = 8.4 Hz, H-1,1′), 7.84 (2H, d, *J* = 8.8 Hz, H-4,5′), 8.15 (2H, d, *J* = 8.8 Hz, H-3,6′), 8.68 (1H, s, *H*C=N), 8.80 (1H, d, *J* = 2.4 Hz, H-5 pyrazine), 8.93 (1H, d, *J* = 2.4 Hz, H-6 pyrazine), 9.27 (1H, s, H-3 pyrazine), 12.32 (1H, s, CON*H*); MS (EI, *m*/*z*): 380 [M^+^], 139, 123, 111, 80, 44.

#### 4-[(E)-(Pyrazine-2-carboylimino)methyl]phenyl 3-bromobenzoate (**4e**)

Colour: white crystals; yield: 0.55 g, 1.3 mmol, 44%; R_f_: 0.5 (40% acetone in *n*-hexane); mp 252–260 °C; IR ($\bar{\nu }$, cm^−1^): 3292, 1734, 1683, 1560, 1249, 1031, 509; ^1^H NMR (400 MHz, DMSO): δ 7.45 (2H, d, *J* = 8.4 Hz, H-2,2′), 7.57–7.61 (1H, m, H-5), 7.84 (2H, d, *J* = 8.4 Hz, H-1,1′), 7.98 (1H, d, *J* = 8 Hz, H-4), 8.14 (1H, d, *J* = 8 Hz, H-6), 8.26 (1H, s, H-3), 8.69 (1H, s, *H*C=N), 8.80 (1H, d, *J* = 2.4 Hz, H-5 pyrazine), 8.93 (1H, d, *J* = 2.4 Hz, H-6 pyrazine), 9.27 (1H, s, H-3 pyrazine), 12.32 (1H, s, CON*H*); MS (EI, *m*/*z*): 426 [M^+^], 183, 157, 123, 104, 80.

#### 4-[(E)-(Pyrazine-2-carboylimino)methyl]phenyl 4-bromobenzoate (**4f**)

Colour: white shiny crystals; yield: 0.91 g, 2.1 mmol, 72%; R_f_: 0.42 (40% acetone in *n*-hexane); mp 295–307 °C; IR ($\bar{\nu }$, cm^−1^): 3286, 1732, 1674, 1585, 1257, 1010, 511; ^1^H NMR (400 MHz, DMSO): δ 7.42 (2H, d, *J* = 8.4 Hz, H-2,2′), 7.84 (4H, d, *J* = 8.4 Hz, H-3,4,5,6), 8.07 (2H, d, *J* = 8.4 Hz, H-1,1′), 8.68 (1H, s, *H*C=N), 8.80 (1H, d, *J* = 2.4 Hz, H-5 pyrazine), 8.93 (1H, d, *J* = 2.4 Hz, H-6 pyrazine), 9.27 (1H, s, H-3 pyrazine), 12.32 (1H, s, CON*H*); MS (EI, *m*/*z*): 426 [M^+^], 183, 157, 123, 104, 80.

#### 4-[(E)-(5-Methylpyrazine-2-carboylimino)methyl]phenyl 2-fluorobenzoate (**4g**)

Colour: Pale yellow powder; yield: 0.48 g, 1.3 mmol, 42%; R_f_: 0.4 (40% acetone in *n*-hexane); mp 250–257 °C; IR ($\bar{\nu }$, cm^−1^): 3304, 1716, 1683, 1560, 1249, 1031, 509; ^1^H NMR (400 MHz, DMSO): δ 2.62 (3H, s, C*H*_*3*_), 7.40–7.44 (4H, m, H-1,1′,2,2′), 7.76–7.84 (3H, m, H-4,5,6), 8.09–8.13 (1H, m, H-3), 8.68 (1H, s, H-6 pyrazine), 8.68 (1H, s, *H*C=N) 9.13 (1H, s, H-3 pyrazine), 12.25 (1H, s, CON*H*); MS (EI, *m*/*z*): 378 [M^+^], 257, 137, 123, 95, 75.

#### 4-[(E)-(5-Methylpyrazine-2-carboylimino)methyl]phenyl 2-chlorobenzoate (**4h**)

Colour: white shiny flakes; yield: 0.94 g, 2.4 mmol, 79%; R_f_: 0.42 (40% acetone in *n*-hexane); mp 213–220 °C; IR ($\bar{\nu }$, cm^−1^): 3296, 1737 (C=O, ester), 1674, 1595 (C=N), 1242, 1197, 1033, 742 (C–Cl aromatic); ^1^H NMR (400 MHz, DMSO): δ 2.63 (3H, s, C*H*_*3*_), 7.43 (2H, d, *J* = 8.4 Hz, H-2,2′), 7.55–7.58 (1H, m, H-5), 7.68–7.69 (2H, m, H-3,4), 7.84 (2H, d, *J* = 8.4 Hz, H-1,1′), 8.11 (1H, d, *J* = 8 Hz, H-6), 8.68 (2H, s, *H*C=N, H-6 pyrazine), 9.11 (1H, s, H-3 pyrazine), 12.27 (1H, s, CON*H*); MS (EI, *m*/*z*): 394 [M^+^], 139, 121, 111, 94, 75.

#### 4-[(E)-(5-Methylpyrazine-2-carboylimino)methyl]phenyl 3-chlorobenzoate (**4i**)

Colour: Lemon green powder; yield: 0.92 g, 2.3 mmol, 77%; R_f_: 0.41 (40% acetone in *n*-hexane); mp 253–260 °C; IR ($\bar{\nu }$, cm^−1^): 3302, 1728, 1678, 1602, 1257, 1199, 1020, 721; ^1^H NMR (400 MHz, DMSO): δ 2.63 (3H, s, C*H*_*3*_), 7.43 (2H, d, *J* = 8.4 Hz, H-2,2′), 7.64–7.68 (1H, m, H-3), 7.82–7.85 (3H, m, H-4,5,6), 8.10 (2H, d, *J* = 8.4 Hz, H-1,1′), 8.68 (2H, s, *H*C=N, H-6 pyrazine), 9.13 (1H, s, H-3 pyrazine), 12.25 (1H, s, CON*H*); MS (EI, *m*/*z*): 394 [M^+^], 139, 121, 111, 94, 75.

#### 4-[(E)-(5-Methylpyrazine-2-carboylimino)methyl]phenyl 4-chlorobenzoate (**4j**)

Colour: white shiny crystals; yield: 0.98 g, 2.5 mmol, 82%; R_f_: 0.45 (40% acetone in *n*-hexane); mp 265–272 °C; IR ($\bar{\nu }$, cm^−1^): 3300, 1734, 1670, 1560, 1261, 1012, 752; ^1^H NMR (400 MHz, DMSO): δ 2.63 (3H, s, C*H*_*3*_), 7.42 (2H, d, *J* = 8.4 Hz, H-2,2′), 7.69 (2H, d, *J* = 8.4 Hz, H-1,1′), 7.82 (2H, d, *J* = 8.4 Hz, H-4,5′), 8.15 (2H, d, *J* = 8.4 Hz, H-3,6′), 8.68 (2H, s, *H*C=N, H-6 pyrazine), 9.13 (1H, s, H-3 pyrazine), 12.24 (1H, s, CON*H*); MS (EI, *m*/*z*): 394 [M^+^], 139, 121, 111, 94, 66.

#### 4-[(E)-(5-Methylpyrazine-2-carboylimino)methyl]phenyl 3-bromobenzoate (**4k**)

Colour: white solid; yield: 0.85 g, 1.9 mmol, 64%; R_f_: 0.42 (40% acetone in *n*-hexane); mp 265–276 °C; IR ($\bar{\nu }$, cm^−1^): 3292, 1732, 1683, 1560, 1247, 1031, 511; ^1^H NMR (400 MHz, DMSO): δ 2.63 (3H, s, C*H*_*3*_), 7.43 (2H, d, *J* = 8.4 Hz, H-2,2′), 7.57–7.61 (1H, m, H-5), 7.83 (2H, d, *J* = 8.4 Hz, H-1,1′), 7.98 (1H, d, *J* = 8 Hz, H-4), 8.14 (1H, d, *J* = 8 Hz, H-6), 8.26 (1H, s, H-3), 8.68 (2H, s, *H*C=N, H-6 pyrazine), 9.14 (1H, s, H-3 pyrazine), 12.25 (1H, s, CON*H*); MS (EI, *m*/*z*): 438 [M^+^], 183, 155, 137, 121, 94.

#### 4-[(E)-(5-Methylpyrazine-2-carboylimino)methyl]phenyl 4-bromobenzoate (**4l**)

Colour: white powder; yield: 0.81 g, 1.8 mmol, 61%; R_f_: 0.5 (40% acetone in *n*-hexane); mp 293–297 °C; IR ($\bar{\nu }$, cm^−1^): 3284, 1735, 1670, 1590, 1261, 1008, 516; ^1^H NMR (400 MHz, DMSO): δ 2.63 (3H, s, C*H*_*3*_), 7.42 (2H, d, *J* = 8.4 Hz, H-2,2′), 7.81–7.85 (4H, m, H-3,4,5,6), 8.07 (2H, d, *J* = 8.4 Hz, H-1,1′), 8.68 (2H, s, *H*C=N, H-6 pyrazine), 9.13 (1H, s, H-3 pyrazine), 12.25 (1H, s, CON*H*); MS (EI, *m*/*z*): 440 [M^+^], 185, 156, 137, 121, 94.

## Results and discussion

**Scheme 1:**
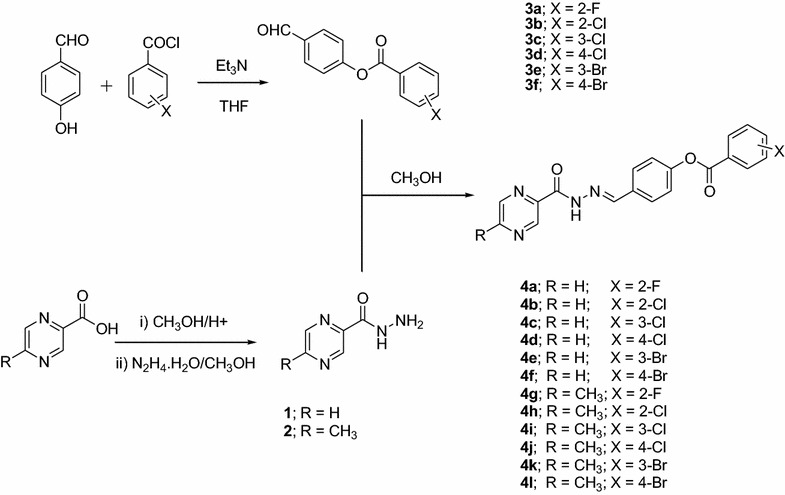
Synthesis of extended iminobenzoates with terminal pyrazine moieties

The target compounds (**4a**–**4l**) were successfully synthesized by reacting hydrazides (**1** and **2**) with the esters (**3a**–**3f**) formed themselves by the reaction of 4-hydroxybenzaldehyde with differently substituted benzoyl chlorides (Scheme 1).

Synthesis of the target compounds was carried out according to scheme 1. Hydrazides **1** and **2** were synthesized following the literature known method [24]. The esters (**3a**–**3f**) were synthesized by reacting 4-hydroxybenzaldehyde with different halogenated benzoyl chlorides in an equimolar ratio. Ranges for the C=O moiety of the ester linkage in the IR spectra of different esters were observed at 1728–1745 cm^−1^ while for its C–O linkage the peaks were noticed at 1253–1265 cm^−1^. Similarly, aldehydic C=O bond displayed the peaks in the range of 1683–1699 cm^−1^ in different esters. C–X (X = halogens) bonds gave their peaks at 513–1207 cm^−1^. Further confirmation to the successful synthesis of the esters was made with NMR studies and the data was consistent with the literature known data [33–35].

The synthesized esters were treated with the hydrazides **1** and **2** in an equimolar ratio resulting in the target iminobenzoates (**4a**–**4l**) in good to excellent yields. Their successful synthesis was confirmed using different spectroanalytical techniques. In the IR spectra, prominent peaks were observed for the NH group of amide linkages in the range of 3284–3304 cm^−1^ while its carbonyl moiety (C=O) displayed peaks in the range of 1670–1683 cm^−1^. The carbonyl group of the ester functionality in different iminobenzoates gave very strong peaks in the range of 1716–1743 cm^−1^. The peaks for the aldehydic moiety were not observed in the final products after being converted to the imine (C=N) group which is also a strong proof for the successful synthesis of the target compounds. Peaks for the new imine functionality were observed in the range of 1560–1610 cm^−1^ in different final products. NMR studies further confirmed the successful synthesis of our target compounds. The proton of the newly formed azomethine (*H*C=N) functionality resonated in the proton NMR spectra in the range of 8.68–8.69 ppm. Similarly, the proton of the amide linkage gave prominent resonance in the range of 12.24–12.36 ppm.

Mass spectra (EIMS) displayed the exact molecular ion peaks for all the synthesized compounds while elemental analysis (Table 1) further aided in the confirmation of the successful synthesis of the target molecules.

**Table 1:** Elemental analyses data of the synthesized final compounds

Compound	Molecular formula	Molecular weight	Calculated (%)			Found (%)		
			C	H	N	C	H	N
**4a**	C_19_H_13_FN_4_O_3_	364.33	62.64	3.60	15.38	62.83	3.78	15.10
**4b**	C_19_H_13_ClN_4_O_3_	380.78	59.93	3.44	14.71	59.64	3.08	14.89
**4c**	C_19_H_13_ClN_4_O_3_	380.78	59.93	3.44	14.71	60.13	3.21	14.93
**4d**	C_19_H_13_ClN_4_O_3_	380.78	59.93	3.44	14.71	60.30	3.70	14.84
**4e**	C_19_H_13_BrN_4_O_3_	425.24	53.67	3.08	13.18	53.58	2.90	13.32
**4f**	C_19_H_13_BrN_4_O_3_	425.24	53.67	3.08	13.18	53.79	3.21	13.35
**4g**	C_20_H_15_FN_4_O_3_	378.36	63.49	4.00	14.81	63.67	4.19	14.69
**4h**	C_20_H_15_ClN_4_O_3_	394.81	60.84	3.83	14.19	60.68	3.59	14.40
**4i**	C_20_H_15_ClN_4_O_3_	394.81	60.84	3.83	14.19	60.72	3.97	14.51
**4j**	C_20_H_15_ClN_4_O_3_	394.81	60.84	3.83	14.19	61.13	4.09	14.01
**4k**	C_20_H_15_BrN_4_O_3_	439.26	54.69	3.44	12.75	54.38	3.35	12.98
**4l**	C_20_H_15_BrN_4_O_3_	439.26	54.69	3.44	12.75	54.82	3.71	12.53

**Fig. 1:**
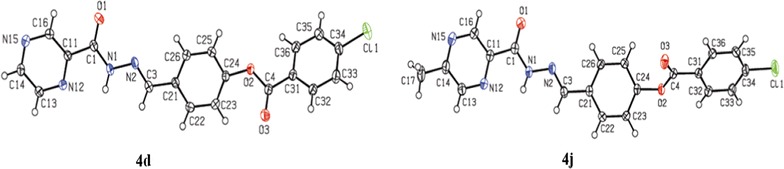
X-ray diffraction structures of compounds **4d** and **4j**

X-ray diffraction analysis stamped well the successful synthesis of the final compounds. Figure 1 and Table 2 shows the XRD structures and main structural parameters of compounds **4d** and **4j**-a further proof to the successful synthesis of these compounds.

**Table 2:** Crystallographic data for compounds **4d** and **4j**

	**4d**	**4j**
Empirical formula	C_19_H_13_ClN_4_O_3_	C_20_H_15_ClN_4_O_3_
Formula weight	380.78	394.81
Temperature (K)	200 (2)	200 (2)
Wavelength (Å)	0.71073	0.71073
Crystal system	Triclinic	Monoclinic
Space group	Pī	P2_1_/c
Unit cell dimensions (Å, ^o^)	a = 5.5960 (3)	a = 22.6336 (8)
	b = 7.3072 (4)	b = 10.9519 (4)
	c = 22.4039 (13)	c = 7.4045 (3)
	α = 95.643 (2)	
	β = 93.132 (2)	β = 97.2090 (10)
	γ = 111.325 (2)	
Volume (Å^3^)	845.21 (8)	1820.93 (12)
Z	2	4
Density (calculated) (Mg/m^3^)	1.496	1.440
Absorption coefficient (mm^−1^)	0.256	0.240
F(000)	392	816
Crystal size (mm^3^)	0.400 × 0.160 × 0.020	0.260 × 0.060 × 0.060
Theta range for data collection (^o^)	1.836 to 30.072	1.814 to 30.115
Index ranges	− 7 ≤ h ≤ 7, − 10 ≤ k ≤ 10,	− 31 ≤ h ≤ 21, − 12 ≤ k ≤ 15,
	− 31 ≤ l ≤ 31	− 10 ≤ l ≤ 10
Reflections collected	15,770	22,479
Independent reflections	4959 [R(int) = 0.0207]	5369 [R(int) = 0.0268]
Absorption correction	Semi-empirical from equivalents	
Max. and min. transmission	0.9949 and 0.9046	0.9857 and 0.9402
Refinement method	Full-matrix least-squares on F^2^	
Data/restraints/parameters	4959/0/244	5369/0/258
Goodness-of-fit on F^2^	1.028	1.027
Final R indices [I > 2σ(I)]	R1 = 0.0392, wR2 = 0.1027	R1 = 0.0413, wR2 = 0.1045
R indices (all data)	R1 = 0.0525, wR2 = 0.1113	R1 = 0.0577, wR2 = 0.1129

**Fig. 2:**
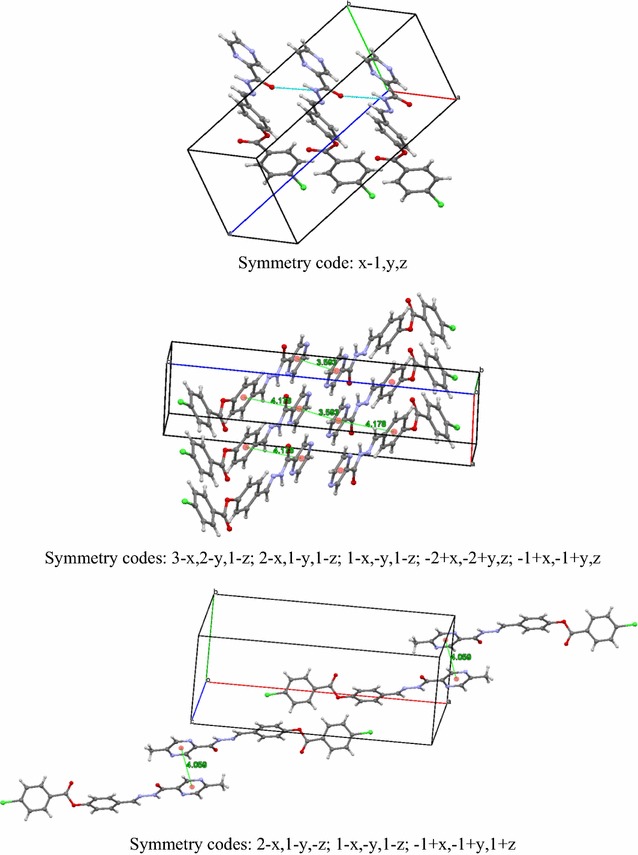
Hydrogen bonding (top) and π–π-stacking interactions (middle) for **4d** and π–π-stacking interactions observed in packing analysis of **4j** (bottom)

Both structures show similar spatial conformation, but with different structural behavior for each side of the central phenyl group (Fig. 1). The pyrazine ring and carbohydrazide system are almost coplanar, with calculated dihedral angles between mean planes of 9.63^o^ and 9.35^o^ for compounds **4d** and **4j**, respectively, and these groups are also coplanar with respect to central phenyl ring. On the other side of the molecule, the dihedral angles between mean planes of central phenyl ring and benzoate moiety is 48.23^o^ for **4d** of 56.25^o^ for **4j**. Packing of **4d** is governed by weak hydrogen bond, which builds a one-dimensional polymeric structure parallel to [100] direction, and by π–π-stacking interactions between two units of neighboring pyrazine rings intercalated by one central phenyl ring, forming a layer parallel to crystallographic plane (Fig. 1). In the case of **4j**, packing is mainly governed π–π-stacking interactions, which were observed between neighboring pyrazine rings forming pairs of molecules related by center of symmetry (Fig. 2).

## Conclusion

The novel iminobenzoates with terminal pyrazine moieties were successfully synthesized while using easily available starting materials. The synthesized compounds were characterized with the help of different spectroanalytical techniques (IR, MS, NMR CHNS, and XRD). The synthesis may provide a useful route to extended π-conjugated systems having central pyrazine moieties in their backbone. Intramolecular charge transfer (ICT) resulted due to the highly π-electron deficient nature of pyrazines would ultimately cause these compounds luminescent. These compounds may also display LC properties if central pyrazines are properly substituted on both the sides.
